# Intra-Arterial Chemotherapy as Primary Treatment for Advanced Unilateral Retinoblastoma in China

**DOI:** 10.3389/fmed.2022.855661

**Published:** 2022-04-07

**Authors:** Tingyi Liang, Xin Zhang, Jiakai Li, Xuming Hua, Peiquan Zhao, Xunda Ji

**Affiliations:** ^1^Department of Ophthalmology, Xinhua Hospital Affiliated to Shanghai Jiao Tong University School of Medicine, Shanghai, China; ^2^Department of Neurosurgery, Xinhua Hospital Affiliated to Shanghai Jiao Tong University School of Medicine, Shanghai, China

**Keywords:** retinoblastoma, advanced stage, unilateral disease, intra-arterial chemotherapy, ophthalmic artery chemosurgery

## Abstract

**Purpose:**

This study aimed to evaluate the efficacy and complications of intra-arterial chemotherapy (IAC) as a primary treatment for advanced unilateral retinoblastoma in Chinese patients.

**Methods:**

This study was a retrospective review of patients with advanced unilateral retinoblastoma treated with IAC as the primary treatment. The IAC procedures were performed using a balloon-assisted technique. The clinical status and treatment complications were recorded at each visit. Kaplan–Meier analysis was performed to estimate recurrence-free survival and ocular survival.

**Results:**

In total, 116 eyes of 116 patients with advanced unilateral retinoblastoma were enrolled, including 66 eyes (57%) in group D and 50 eyes (43%) in group E. All treated eyes received a mean of 3 cycles of IAC (range, 3–5), and 66% of the eyes were combined with local consolidation therapy. The median follow-up time was 39 months (range, 22–57 months). The 3-year recurrence-free survival and ocular survival rates were 68.8% (95% CI, 59.2–76.6%) and 88.5% (95% CI, 80.9–93.2%), respectively. Moreover, the 3-year ocular survival rate in group D was significantly higher than that in group E (96.9%, 76.3%; *P* < 0.01). The common ocular complication was vitreous hemorrhage (19.8%). No deaths or severe systemic complications occurred.

**Conclusion:**

Primary intra-arterial chemotherapy is effective for the treatment of advanced unilateral retinoblastoma, especially in group D, with acceptable toxicity.

## Introduction

Retinoblastoma is the most common primary intraocular malignant tumor in children (1). Over the past three decades, multiple treatment modalities have been applied for the management of retinoblastoma without the need for external beam radiation or enucleation. Since the mid-1990s, intravenous chemotherapy (IVC) has become the most commonly used treatment for retinoblastoma and achieved treatment success rates ranging from 90 to 100% for early-stage retinoblastoma (groups A–C) (2). However, IVC alone is rarely curative for eyes with advanced retinoblastoma (groups D and E), and the treatment success rates are less than 50% (2–4). Additionally, the systemic adverse effects associated with IVC, such as secondary acute myelogenous leukemia, are worrisome (5).

Intra-arterial chemotherapy (IAC), also known as ophthalmic artery chemosurgery (OAC), has been introduced in the management of retinoblastoma for several years (6–9). Compared with IVC, IAC directly delivers chemotherapy drugs into the eye, showing substantial advantages in increasing intraocular tumor exposure to the drugs and reducing systemic adverse effects. IAC allows many eyes with advanced retinoblastoma to be saved; previously, these eyes would have required enucleation (10). Due to the remarkable treatment effect, many centers worldwide prefer to use IAC for the treatment of retinoblastoma, especially advanced retinoblastoma (11).

Currently, there are no clear clinical guidelines regarding whether IAC can be used as a primary treatment for advanced retinoblastoma; clinicians mainly make treatment choices based on their experience. In the present study, we evaluated the efficacy and complications of IAC as the primary treatment for advanced unilateral retinoblastoma in a Chinese population. We hope that this study will serve as a powerful reference for developing clinical guidelines in the management of advanced unilateral retinoblastoma.

## Materials and Methods

### Patients

This was a single-center, single-arm retrospective study. The study was approved by the Ethics Committee of Xinhua Hospital Affiliated to Shanghai Jiao Tong University School of Medicine and adhered to the tenets of the Declaration of Helsinki. Written informed consent was obtained from the guardian of each patient. The medical records of all patients diagnosed with advanced unilateral retinoblastoma and treated at Xinhua Hospital Affiliated to Shanghai Jiao Tong University School of Medicine between January 2016 and December 2018 were reviewed. The inclusion criteria were patients with advanced unilateral retinoblastoma who received IAC as the primary treatment. Advanced retinoblastoma was defined as International Intraocular Retinoblastoma Classification (IIRC) (12) groups D and E (Table 1). Patients were excluded if they (1) received previous treatment before referral to our hospital; (2) had high-risk retinoblastoma with tumor invasion into the anterior chamber, choroid or retrolaminar optic nerve; or (3) had extraocular tumor extension or metastatic disease.

**Table 1:** International intraocular retinoblastoma classification (IIRC).

Group A	• Small (< 3 mm) discrete tumor at least 3 mm from foveola and 1.5 mm from optic nerve. • No vitreous or subretinal seeding.
Group B	• Eyes with no vitreous or subretinal seeding and discrete retinal tumor of any size and location. • Subretinal fluid < 5 mm from the base of the tumor.
Group C	• Eyes with only focal vitreous or subretinal seeding and discrete retinal tumor of any size and location. • Subretinal fluid < 1 quadrant.
Group D	• Eyes with diffuse vitreous or subretinal seeding and/or massive non-discrete endophytic or exophytic disease. • Eyes with more extensive seeding than Group C. Massive and/or diffuse intraocular disseminated disease may consist of fine or greasy vitreous seeding or avascular masses. Subretinal seeding may be plaque like. Included exophytic disease and more than one quadrant of retinal detachment.
Group E	Eyes anatomically or functionally destroyed, including irreversible neovascular glaucoma, massive intraocular hemorrhage, aseptic orbital cellulitis, tumor anterior to anterior vitreous face, tumor touching the lens, diffuse infiltrating retinoblastoma, phthisis bulbi or pre-phthisis.

### Treatment

The IAC procedure was based on the Japanese balloon-assisted IAC infusion method (13). IAC was performed under general anesthesia and systemic intravenous heparinization. After femoral artery puncture using the Seldinger technique, a 5-French (5-F) catheter was introduced into the ipsilateral internal carotid artery under fluoroscopic guidance. Then, a microballoon catheter was passed through the introducer catheter and moved beyond the orifice of the ophthalmic artery (OA). Temporary occlusion was achieved by inflating the balloon. After fluoroscopic confirmation of balloon occlusion at the portion immediately distal to the orifice of the OA, chemotherapy drugs were injected from the introducer catheter and infused into the OA. The chemotherapy agents included carboplatin (30–60 mg), topotecan (1 mg) and melphalan (3–7 mg). The specific drug dosage was determined based on the patient's age and severity of the disease. The dosage of melphalan should be less than 0.5 mg/kg. IAC was performed at monthly intervals. As needed, local consolidation therapy, including thermotherapy, laser photocoagulation, cryotherapy and intravitreal chemotherapy, was used for tumor consolidation in conjunction with or after the completion of IAC. Thermotherapy was performed for small retinal tumors (<3 mm), and laser photocoagulation was performed for slightly larger retinal tumors by surrounding the tumor and closing off feeding vessels. Cryotherapy was mainly used for peripheral tumors. Intravitreal chemotherapy was performed for vitreous seeds control.

### Follow-Up

The patients underwent monthly comprehensive ocular examinations, including anterior segment evaluation, fundus examination with indirect ophthalmoscopy, fundus photography, B-scan ultrasonography, and, as needed, fluorescein fundus angiography (FFA). The clinical status and complications were recorded at each visit. If treatment success was achieved, the follow-up time was extended to 3 and 4 months, and then every 6 months thereafter. Treatment success was defined as the complete regression of the intraocular tumor without recurrence. Recurrent disease was defined as regrowth of inactive tumor or seeds that required further treatment. Meanwhile, the patients were followed up with orbit/brain MRI every 6 months for systemic monitoring.

### Statistical Analysis

The statistical analysis was performed with Prism (GraphPad Software, La Jolla California USA). Kaplan–Meier analysis was performed to estimate recurrence-free survival and ocular survival, and the Mantel–Cox test was used to compare the survival curves. A *P* < 0.05 was considered statistically significant.

## Results

In total, 116 eyes of 116 consecutive patients with advanced unilateral retinoblastoma were included (Table 2). The average age at diagnosis was 22 months (range, 6–120 months), and 61 patients (53%) were male. According to the IIRC, 116 treated eyes were classified as group D (*n* = 66, 57%) and group E (*n* = 50, 43%). The mean follow-up time was 38 months (median, 39 months; range, 22–57 months).

**Table 2:** Patient characteristics.

**Features**	**No. (%)**
Treated patients	116
Treated eyes	116
Mean age (median, range), mos	22 (12, 6–120)
Sex	
Male	61 (53%)
Female	55 (47%)
Eye	
Right	56 (48%)
Left	60 (52%)
IIRC	
Group D	66 (57%)
Group E	50 (43%)
IAC cycles, mean (median, range)	3 (3, 3–5)
Local therapy	
With	76 (66%)
Without	40 (34%)
Mean follow-up (median, range), mos	38 (39, 22–57)

*IIRC, International Intraocular Retinoblastoma Classification; IAC, intra-arterial chemotherapy*.

**Figure 1:**
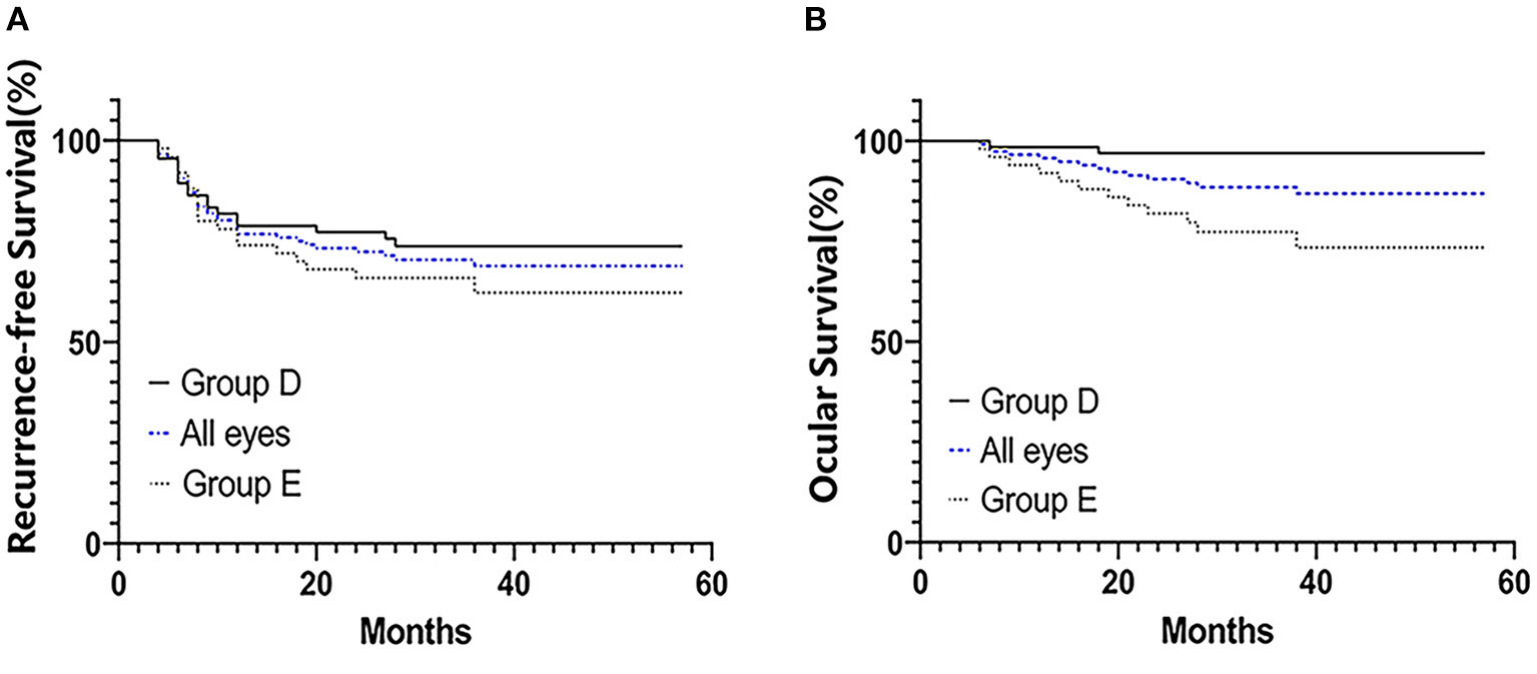
Kaplan–Meier survival curves for 116 patients with advanced unilateral retinoblastoma who were treated with primary IAC treatment. Recurrence-free survival **(A)** and ocular survival **(B)**. Patients' eyes were divided into groups according to the IIRC.

**Figure 2:**
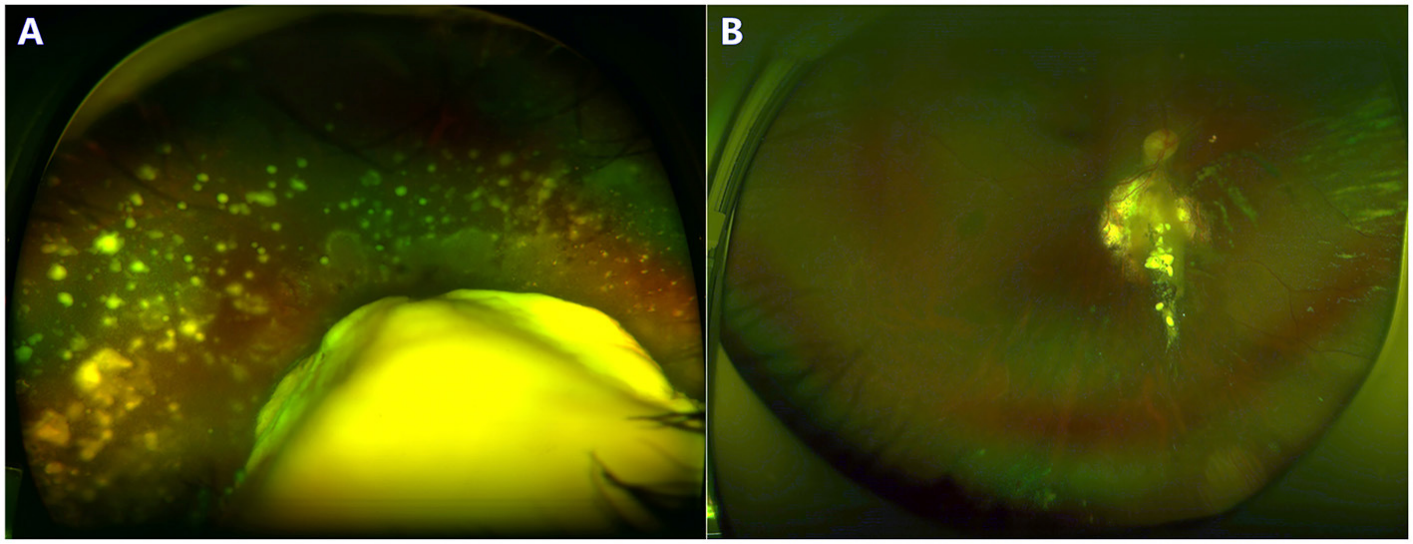
A 3-year-old boy with group D retinoblastoma presented with a large retinal tumor and massive subretinal seeds. The regression of tumor and seeds was noted at the 24-month follow-up. Before **(A)** and after **(B)** 3 cycles of primary IAC infusion and laser consolidation treatment.

**Figure 3:**
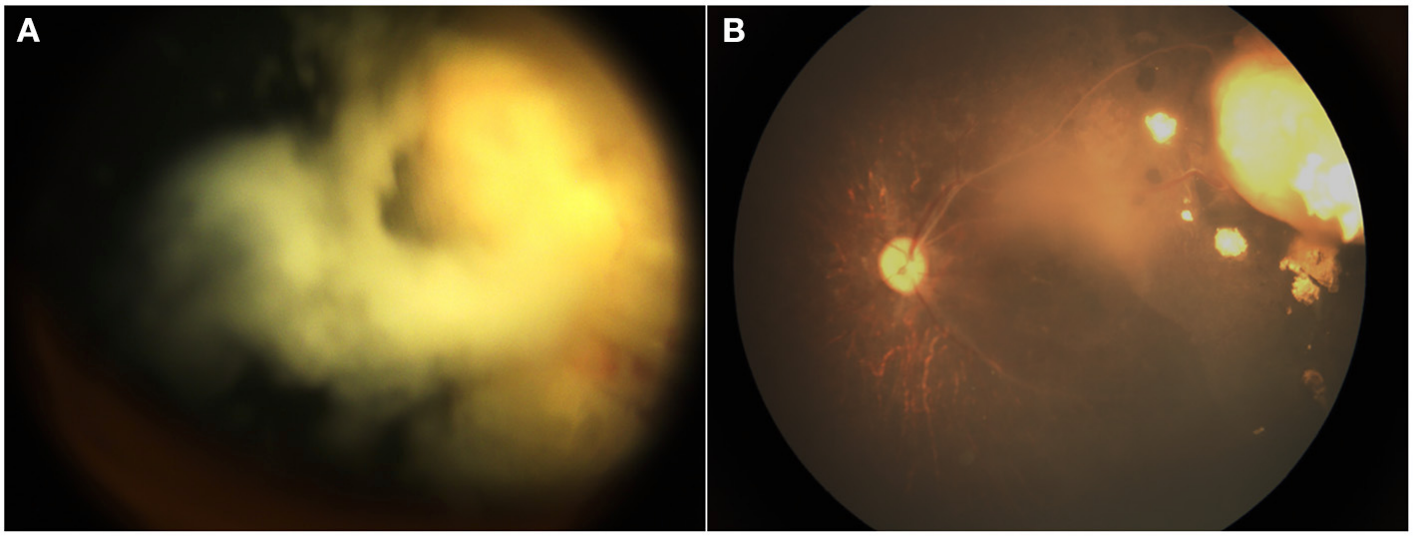
A 12-month-old boy with group D retinoblastoma presented with a peripheral retinal tumor and diffuse vitreous seeds. The regression of tumor and seeds was noted at the 18-month follow-up. Before **(A)** and after **(B)** 3 cycles of primary IAC infusion combined with intravitreal chemotherapy.

The treated eyes received no prior treatment and initially received an average of 3 cycles of IAC (range, 3–5). The IAC procedures were technically successful in all eyes. In addition, 76 eyes (66%) received local therapy combined with IAC: thermotherapy and/or laser photocoagulation (*n* = 27), cryotherapy (*n* = 20) and intravitreal chemotherapy (*n* = 43). Treatment success was achieved in 81 eyes (69.8%); tumor recurrence occurred in 35 eyes (30.2%) within an average of 11 months (range, 4–36 months) after completing primary treatment. Further treatment for recurrent disease was performed as follows: additional IAC (*n* = 19), local therapy (*n* = 22), and enucleation (*n* = 7). The 36-month overall recurrence-free survival rate was 68.8% (95% CI, 59.2–76.6%) and 73.7% (95% CI, 61.2–82.8%) in group D and 62.2% (95% CI, 46.3–74.6%) in group E (*P* = 0.266; Figure 1A). At the final follow-up, globe salvage was achieved in 106 eyes (87%). Figures 2, 3 show representative cases in the present study. The 36-month overall ocular survival rate was 88.5% (95% CI, 80.9–93.2%) and 96.9% (95% CI, 88.4–99.2%) in group D and 77.3% (95% CI, 62.7–86.8%) in group E (*P* = 0.0006; Figure 1B). The causes for enucleation included persistent dense vitreous hemorrhage and/or neovascular glaucoma (NVG) (*n* = 8) and tumor recurrence (*n* = 7). Pathological reports showed that the resection margins and optic nerve were tumor-free. One patient developed bone metastasis 6 months after enucleation and was subsequently treated with aggressive intravenous chemotherapy, achieving disease control. No deaths occurred (100% patient survival rate).

The details of the treatment complications are shown in Table 3. The most common ocular complication was vitreous hemorrhage (*n* = 23; 19.8%). Mild vitreous hemorrhage was observed in 15 eyes and was self-absorbed; however, the other 8 eyes, which showed persistent dense vitreous hemorrhage and NVG, lost the control of the fundus and received enucleation. Other ocular complications included lens opacity (*n* = 11; 9.5%), chorioretinal atrophy (*n* = 4; 3.4%), vitreoretinal fibrosis (*n* = 2; 1.7%), rhegmatogenous retinal detachment (RRD) (*n* = 1; 0.9%) and phthisis bulbi (*n* = 1; 0.9%). No severe systemic complications occurred. The main systemic complications included transient leukopenia (*n* = 41; 35.3%) and vomiting (*n* = 21; 18.1%).

**Table 3:** Treatment complications.

**Complications**	**No. (%)**
Ocular	
Vitreous hemorrhage	23 (19.8%)
Lens opacity	11 (9.5%)
Chorioretinal atrophy	4 (3.4%)
Vitreoretinal fibrosis	2 (1.7%)
RRD	1 (0.9%)
Phthisis bulbi	1 (0.9%)
Systemic	
Transient leukopenia	41 (35.3%)
Vomiting	21 (18.1%)
Rash	6 (5.1%)
Epistaxis	4 (3.4%)
Periocular erythema	3 (2.6%)
Fever	2 (1.7%)

*RRD, rhegmatogenous retinal detachment*.

## Discussion

In the present study, we reported our experience performing IAC as the primary treatment for 116 Chinese patients with advanced unilateral retinoblastoma. Our study confirms that the majority of advanced unilateral retinoblastoma could be successfully managed with primary IAC, avoiding the need for enucleation. In our study, a high rate of ocular salvage was achieved with an overall success rate of 87% (*n* = 101/116). The 36-month ocular survival rate in group D was dramatically 96.9%, which was significantly higher than that in group E (77.3%; *P* < 0.01). The results of our study are consistent with the data reported by other researchers. Shields et al. reported that primary IAC provided superior globe salvage rates for unilateral retinoblastoma of 91 and 48% in groups D and E, respectively (14). Abramson et al. showed successful IAC treatment for naïve advanced retinoblastoma with a 5-year ocular survival rate of 80.2% (10, 15). In another study focusing on group D retinoblastoma, the globe salvage rate at 110 months with primary IAC was 85% (16). According to the work by Munier and colleagues, 25 patients with unilateral group D retinoblastoma received primary IAC, and after a mean follow-up of 41.7 months, all treated eyes (100%) were saved with no need for enucleation (8). An opinion report from the above three major IAC centers agreed with the preference for IAC for the treatment of advanced unilateral retinoblastoma (17). Therefore, it seems that primary IAC shows superiority in the management of advanced unilateral retinoblastoma, achieving a globe salvage rate of approximately 90%.

Since Reese first reported the use of IAC for the treatment of retinoblastoma in 1958 (18), IAC technology has been continuously developed. Japanese investigators developed a technique referred to as “selective ophthalmic artery infusion (SOAI)” with a balloon catheter, which temporarily occludes the internal carotid artery (distal to the OA orifice) and allows chemotherapy drugs to be infused into the OA (13). More recently, Abramson et al. introduced another SOAI technique, superselective OA catheterization, involving a microcatheter directly introduced into the OA through which high doses of chemotherapy drugs could be directly administered into the eye (6, 19). However, compared with the Japanese method, the treatment failure rate of superselective OA catheterization was higher due to the technical difficulty of inserting the microcatheter into the OA, especially in an infant; moreover, there was a higher risk of damage to the vascular intima during OA catheterization, which could lead to a series of ophthalmic vascular events (20). In our study, the average age of the treated patients was young (mean, 22 months), and in our experience, East Asians generally grow slowly with relatively narrow ophthalmic arteries, which may increase the difficulty of superselective OA catheterization. We adopted the Japanese balloon-assisted IAC infusion method; as a result, technical success of IAC procedures was achieved in all treated eyes, and we did not experience serious adverse events associated with IAC.

In our study, all eyes were primarily treated with an average of 3 cycles of IAC infusion, which resulted in significant intraocular tumor shrinkage and regression. Meanwhile, the treated eyes also benefited from additional local consolidation therapies for tumor control. Combined with chemoreduction, local therapy including thermotherapy, laser photocoagulation and cryotherapy were beneficial for retinal tumor control (21); in addition, intravitreal chemotherapy was particularly effective in treating vitreous seeds, which commonly resulted in treatment failure with IAC alone in eyes with severe vitreous disease (22). Recurrent disease was observed in 35 (30.2%) treated eyes, while we did not observe a difference in the recurrence rate between group D and group E (*P* = 0.266). Seven eyes were enucleated due to recurrent disease, while the other eyes with recurrence achieved tumor control with additional IAC and/or local therapies. In addition, 8 eyes were enucleated due to persistent dense vitreous hemorrhage and NVG. Considering the continuous development of eye-preserving management of advanced retinoblastoma, secondary neovascularization may become an important cause of globe salvage failure (23, 24). The exact causes of neovascularization in a treated eye with advanced retinoblastoma may be complicated, not only related to treatment side effects but also related to the malignancy of the tumor, tumor progression/recurrence, chronic retinal detachment, and other factors (23). In our opinion, we recommended early enucleation in cases that lost the control of the fundus due to fear of neovascularization concomitant with active tumor.

With the widespread use of IAC, it was noted that the outstanding tumor response was followed by IAC-related ophthalmic vascular events, including chorioretinal ischemia, ophthalmic artery occlusion, intraocular hemorrhage, retinal artery/vein occlusion and others (25–29). The exact causes of these vascular complications remain undetermined, which may be due to vascular injury during IAC infusion or chemotherapy drug-mediated toxicity (26). Chorioretinal ischemia, also called chorioretinal occlusive vasculopathy or chorioretinal atrophy, was the most frequently reported vascular complication, developing in 7% (28, 29) to 47% (25, 30) of the eyes. In our study, chorioretinal atrophy was observed in 3.4% of the treated eyes; the low incidence may be attributed to the balloon-assisted technique, which eliminates the need for OA catheterization and thus avoids direct vascular injury. Vitreous hemorrhage was commonly observed in 19.8% of the eyes in the present study; except for 7 cases that showed persistent dense vitreous hemorrhage and NVG and required enucleation, the other cases showed mild vitreous hemorrhage, which was self-absorbed. We speculate that the occurrence of vitreous hemorrhage is not entirely due to IAC but also includes malignancy of the intraocular tumor (all advanced stage), retinal detachment, and side effects of tumor regression. In addition, we did not observe ophthalmic artery occlusion, which is considered a serious vision-threatening complication (28). During the follow-up time, severe life-threatening complications, such as cerebrovascular accident, retinal failure, or secondary cancer, were not observed. It seems that primary IAC with the balloon-assisted technique is a safe treatment modality for advanced retinoblastoma, achieving effective tumor control with acceptable toxicity.

Although primary IAC achieves a high globe salvage rate in advanced unilateral retinoblastoma, patient survival must not be compromised. In our study, no deaths occurred until the final follow-up; however, one patient developed bone metastases and received aggressive systemic chemotherapy, achieving tumor control. Theoretically, IAC had minimal systemic effects and would likely be insufficient in preventing metastatic disease. However, several studies mentioned below have reported that the use of IAC did not increase the risk of metastatic deaths. A comparative study found that there was no significant difference in the metastatic mortality rate between patients treated with primary IAC and those treated with IAC plus IVC (3 vs. 7%; *P* = 0.51) for advanced retinoblastoma; additional IVC combined with IAC did not show superiority in systemic control (31). A report from four major IAC centers worldwide showed that the use of IAC did not increase metastasis or metastasis-related deaths (17). Another worldwide report (*n* = 1,139) demonstrated that the risk of metastatic deaths in IAC-treated patients was <0.01 (32). It seems that the timely and effective control of intraocular retinoblastoma is the key to treatment and can reduce the risk of metastasis; however, high-risk retinoblastoma presenting with choroid or retrolaminar optic nerve invasion should be treated with primary enucleation and postoperative adjuvant chemotherapy to avoid metastatic disease.

This study had several limitations. First, this is a single-center retrospective study. Second, this study lacks a control group to evaluate the true benefit of primary IAC for advanced unilateral retinoblastoma. However, the rarity of retinoblastoma limited the present study to a retrospective, non-comparative cases series. A multicenter, randomized controlled trial will be valuable in the future. Last but not least, we reported 3-year survival outcomes, which may not be sufficient to represent long-term success, and it is necessary to determine 5-, 10-, and 15-year survival rates to assess long-term survival outcomes in these patients.

In summary, the findings from our relatively large cohort confirm the superiority of primary IAC for advanced unilateral retinoblastoma with acceptable ocular toxicity and minimal systemic adverse effects. For group D unilateral retinoblastoma, we strongly recommend that IAC should be the preferred treatment modality, achieving an impressive 3-year survival rate of 96.9% in the present study. We believe our study can serve as a powerful reference for clinicians' decision making in the future.

## Data Availability Statement

The original contributions presented in the study are included in the article/supplementary material, further inquiries can be directed to the corresponding authors.

## Ethics Statement

The studies involving human participants were reviewed and approved by the Ethics Committee of the Xinhua Hospital Affiliated to Shanghai Jiao Tong University School of Medicine. Written informed consent to participate in this study was provided by the participants' legal guardian/next of kin.

## Author Contributions

XJ, PZ, and XH: conception and design. TL, XZ, and JL: collection and assembly of data. All authors contributed to the production of this manuscript and approved the final version.

## Funding

This study was supported by the Clinical Research Plan of SHDC (Grant No: SHDC2020CR1009A) and Hospital Funded Clinical Research Plan of the Xinhua Hospital Affiliated to Shanghai Jiao Tong University School of Medicine (Grant No: 21XHDB08).

## Conflict of Interest

The authors declare that the research was conducted in the absence of any commercial or financial relationships that could be construed as a potential conflict of interest.

## Publisher's Note

All claims expressed in this article are solely those of the authors and do not necessarily represent those of their affiliated organizations, or those of the publisher, the editors and the reviewers. Any product that may be evaluated in this article, or claim that may be made by its manufacturer, is not guaranteed or endorsed by the publisher.
